# Association of predicted fat mass and lean body mass with diabetes: a longitudinal cohort study in an Asian population

**DOI:** 10.3389/fnut.2023.1093438

**Published:** 2023-05-09

**Authors:** Maobin Kuang, Song Lu, Ruijuan Yang, Huaigang Chen, Shuhua Zhang, Guotai Sheng, Yang Zou

**Affiliations:** ^1^Medical College of Nanchang University, Nanchang, Jiangxi, China; ^2^Jiangxi Cardiovascular Research Institute, Jiangxi Provincial People’s Hospital, The First Affiliated Hospital of Nanchang Medical College, Nanchang, Jiangxi, China; ^3^Department of Endocrinology, Jiangxi Provincial People’s Hospital, The First Affiliated Hospital of Nanchang Medical College, Nanchang, Jiangxi, China; ^4^Jiangxi Provincial Geriatric Hospital, Jiangxi Provincial People’s Hospital, The First Affiliated Hospital of Nanchang Medical College, Nanchang, Jiangxi, China

**Keywords:** predicted fat mass, protective factors, predicted lean body mass, diabetes, risk management

## Abstract

**Objective:**

The relationship between body composition fat mass (FM) and lean body mass (LBM) and diabetes risk is currently debated, and the purpose of this study was to examine the association of predicted FM and LBM with diabetes in both sexes.

**Methods:**

The current study was a secondary analysis of data from the NAGALA (NAfld in the Gifu Area, Longitudinal Analysis) cohort study of 15,463 baseline normoglycemic participants. Predicted LBM and FM were calculated for each participant using anthropometric prediction equations developed and validated for different sexes based on the National Health and Nutrition Examination Survey (NHANES) database, and the outcome of interest was diabetes (types not distinguished) onset. Multivariate Cox regression analyses were applied to estimate the hazard ratios (HRs) and 95% confidence intervals (CIs) for the associations of predicted FM and LBM with diabetes risk and further visualized their associations using a restricted cubic spline function.

**Results:**

The incidence density of diabetes was 3.93/1000 person-years over a mean observation period of 6.13 years. In women, predicted LBM and FM were linearly associated with diabetes risk, with each kilogram increase in predicted LBM reducing the diabetes risk by 65% (HR 0.35, 95%CI 0.17, 0.71; *P* < 0.05), whereas each kilogram increase in predicted FM increased the diabetes risk by 84% (HR 1.84, 95%CI 1.26, 2.69; *P* < 0.05). In contrast, predicted LBM and FM were non-linearly associated with diabetes risk in men (all *P* for non-linearity < 0.05), with an L-shaped association between predicted LBM and diabetes risk and a saturation point that minimized the risk of diabetes was 45.4 kg, while predicted FM was associated with diabetes risk in a U-shape pattern and a threshold point with the lowest predicted FM-related diabetes risk was 13.76 kg.

**Conclusion:**

In this Asian population cohort, we found that high LBM and low FM were associated with lower diabetes risk according to anthropometric equations. Based on the results of the non-linear analysis, we believed that it may be appropriate for Asian men to keep their LBM above 45.4 kg and their FM around 13.76 kg.

## Introduction

Diabetes, characterized by chronically elevated blood glucose, is a cluster of metabolic disorders due to impaired insulin secretion or insulin resistance (IR) (1, 2). The number of patients with diabetes has quadrupled over the past thirty years and is expected to grow to 693 million by 2045 consequent upon the aging and changes in diet structure and lifestyle of the global population (3, 4). The dangers of diabetes are not limited to disorders of blood glucose metabolism, but can also cause acute and chronic complications in multiple organ systems such as the heart, retina, kidneys, and brain and is the main reason for disability and premature death (5–7). Therefore, early detection and intervention of risk factors for diabetes can greatly reduce the burden on national healthcare systems and have important public health implications.

Obesity is a well-recognized risk factor for diabetes, and body mass index (BMI) is the most commonly used anthropometric parameter to evaluate obesity. However, the limitation that BMI cannot distinguish between fat and lean muscle mass makes it of very limited help in preventing diabetes (8). In recent years, more and more studies have demonstrated that body composition indicators FM and LBM can reflect more obesity-related clinical and public health information than BMI, and that in-depth analysis of the different contributions of FM and LBM to BMI can help explain the obesity paradox (9–11); in addition, FM and LBM have significant but distinct effects on human glucose metabolism (12, 13). Therefore, we speculated that exploring the independent effects of LBM and FM in relation to diabetes risk beyond BMI may improve our understanding of the association of diabetes risk with BMI, and can provide more accurate reference data for diabetes prevention and treatment. Several related studies have concluded that FM was positively correlated with diabetes risk and the correlation was stronger than that of BMI (10, 14, 15); however, there are some debates about the results of the independent association between LBM and diabetes risk (14, 16–21). For example, Haines et al. concluded that low skeletal muscle mass independent of body fat distribution was associated with an elevated risk of diabetes in men, but not in women (18); whereas the study by Colpitts et al. found no increased risk of diabetes in participants with high BMI and low LBM compared to those with high BMI and high LBM, therefore, they placed more emphasis on the importance of BMI in the risk of diabetes (19), and similarly, the study by Baker et al. showed a non-significant relationship between LBM and diabetes risk (14); additionally, Liu et al. and Rehunen et al. suggested that LBM may be a risk factor for diabetes and an independent predictor of the onset of diabetes in men (16, 17). These differences may be due to different study designs, study populations and sample sizes, and the way in which LBM was measured. Consequently, in order to clarify the independent association between body compositions and diabetes risk in Asian populations, based on the large longitudinal cohort of the NAGALA study (*N* = 15,463), this study further explored and compared the associations between risk for future diabetes and BMI and FM and LBM. Furthermore, due to the high economic and technical costs of dual-energy X-ray absorptiometry (DXA), the gold standard measure of body composition LBM and FM, it is generally used for laboratory examinations rather than for large-scale population measurements. Therefore, consistent with previous studies (16), the LBM and FM in the current study were also calculated using the sex-specific anthropometric prediction equations developed and validated by Lee DH et al. based on the NHANES database (22).

## Materials and methods

### Study design

This study used data from the NAGALA cohort study from 1994 to 2016. The cohort study was initiated in 1994 and investigators continuously recruited and followed up with participants who underwent health screening at Murakami Memorial Hospital to evaluate chronic diseases such as diabetes and non-alcoholic fatty liver and their risk factors that affect the health of the general population (23). Data from the NAGALA cohort had been uploaded to the Dryad database by Professor Okamura (24). We used the data to explore the relationship between predicted FM and LBM and BMI and the risk of diabetes without violating the terms of the database.

### Study population and ethical approval

**FIGURE 1:**
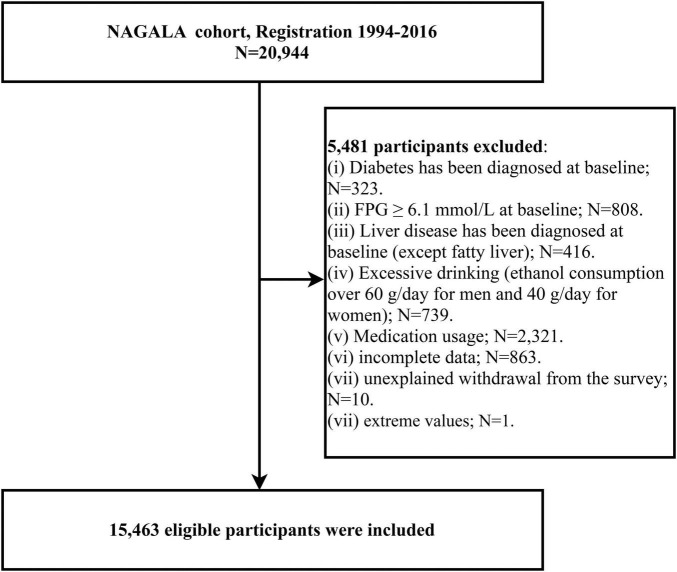
Flowchart of the selection process of study participants.

We extracted data from 12,498 men and 8,446 women who enrolled in the NAGALA cohort. The follow-up period for each subject was calculated from the date of enrollment in the cohort until the onset of diabetes or missed visits or the study cut-off date, whichever occurred first, using 31 December 2016, as the follow-up cut-off date. For the purposes of the current study, we excluded participants with the following characteristics: incomplete baseline information (*n* = 863) and data with extreme values (*n* = 1), fasting plasma glucose (FPG) ≥ 6.1 mmol/L (*n* = 808) or diagnosis of diabetes at baseline examination (*n* = 323), taking any medication at baseline (*n* = 2321), diagnosed liver disease at baseline (except fatty liver) (*n* = 416), excessive alcohol consumption (25) (*n* = 739), and unexplained withdrawal from the follow-up cohort (*n* = 10). Figure 1 shows the detailed inclusion and exclusion criteria.

The NAGALA cohort study has been ethically reviewed by the Ethics Committee of Murakami Memorial Hospital, and after the eligible participants have enrolled, the use of their data was explained to them, and then they signed a written informed consent (IRB 2018-09-01). As a secondary analysis of NAGALA data, all procedures for this study are in line with the Helsinki Declaration, see STROBE statement (Supplementary text), and are approved by the Ethics Committee of Jiangxi Provincial People’s Hospital (IRB 2021-066).

### Exposure variables

The predicted FM and LBM in this study were calculated using the anthropometric prediction equations developed and validated by Lee et al., and the detailed steps for the construction of the equations have been described elsewhere (22). Briefly, Lee et al. extracted the data of 6,534 women and 7,531 men who had the measurements of DXA from the NHANES database, and then used the DXA-measured FM and LBM as dependent variables, and used simple anthropometric and demographic information of the participants as independent variables to develop linear prediction models; after repeated model fitting and then validating in another independent cohort it was found that FM and LBM predicted by the linear regression models with age, height, race, waist circumference (WC) and weight as independent variables had the highest agreement with FM and LBM measured by DXA [FM (man: *R*^2^ = 0.90; woman: *R*^2^ = 0.93)] and [LBM (man: *R*^2^ = 0.91; woman: *R*^2^ = 0.85)]. Among these predictors, the race variable was a categorical variable that was divided into four categories: Mexican, Hispanic, Black, and Other races, and different race categories were assigned different values in the equations; the Asian population in the current study belongs to Other races, so the assignment values of the race variable for the current study population were −1.007 and 1.050 in the prediction equations for LBM and FM for men, respectively, and −0.34 and 0.325 in the prediction equations for LBM and FM for women, respectively. We calculated the predicted LBM and FM for each participant using the equations presented in Supplementary Table 1. BMI was obtained by weight (kg)/[height (m)]^2^.

### Other variables

Participants self-reported information on sex, age, exercise habits, smoking status, and drinking status in a standardized questionnaire. Regarding lifestyle habits, participants who had more than one physical activity per week were considered to have exercise habits; smoking status was defined as none or past or current according to whether subjects smoked in the past or currently; participants’ drinking status was defined as non/small or light or moderate or heavy depending on the type and amount of alcohol they consumed per week during the month before the initial visit (25). Participants wearing no shoes and with light clothing indoors had their WC, systolic/diastolic blood pressure (SBP/DBP), weight, and height measured by medical examiners according to standard methods. In addition, after the ultrasound professional technicians collected the abdominal ultrasound images of the participants, fatty liver was diagnosed by a gastroenterologist without knowing any other information about the participant based on four known image characteristics (liver brightness, deep attenuation, hepatorenal echo contrast, and vascular blurring), in which participants with liver brightness and hepatorenal echo contrast characteristics were diagnosed as having fatty liver (26).

Participants’ forearm venous blood samples at fasting (at least 8 h) were drawn by professional medical examiners and then used standard procedures to measure the concentration of biochemical parameters including total cholesterol (TC), γ-glutamyltransferase (GGT), glycosylated hemoglobin (HbA1c), low-density lipoprotein cholesterol (LDL-C), alanine aminotransferase (ALT), FPG, high-density lipoprotein cholesterol (HDL-C), aspartate aminotransferase (AST), and triglyceride (TG).

### Outcome: diabetes onset

Diagnostic criteria for the onset of diabetes include: (1) normoglycemia at baseline but self-reported diabetes diagnosis during follow-up (verified by the investigators through reviewing of the participant’s medical records and glucose measurement records); (2) normoglycemia at baseline but measured FPG ≥ 7.0 mmol/L or HbA1c ≥ 6.5% during follow-up (27).

### Statistical analysis

Data analyses were carried out by R language version 3.4.3 and Empower (R) version 2.0, with a *P* value < 0.05 (bilateral) as the significance criterion; in addition, as the large gender differences in body composition, all analyses were conducted separately in women and men (28). Baseline data of participants were grouped according to whether they were developing diabetes, and measurement data were presented as median (interquartile range) or mean (standard deviation) and used the Mann–Whitney U test or Student’s *t*-test to compare the inter-group difference; count data were presented as frequency (%) and used the chi-square test to compare the inter-group difference. Additionally, the inverse probability of treatment weighting method was further used to calculate weighted standardized differences between groups (differences > 10% were considered significant) for visually describing the magnitude of differences between groups (29). Before conducting association analysis, variables were examined first for multicollinearity using a variance inflation factor (VIF) and were excluded if VIF > 5. According to the recommendations of the STROBE statements (30), we constructed four multivariate time-dependent Cox proportional hazard regression models for estimating HRs and 95% CIs of the associations between the predicted FM and LBM and BMI and the risk of diabetes. In model 1, age and height were first adjusted; based on model 1, model 2 considered the effects of smoking status, exercise habits, and drinking status on diabetes risk; model 3 was further adjusted for TC, ALT, TG, SBP, AST, HDL-C, HbA1c, GGT, FPG based on model 2; finally, to minimize residual confounding we included all non-collinear covariates into model 4. It is worth mentioning that to assess the independent effects of predicted FM and LBM on diabetes risk, we adjusted the two for each other in all models. In addition, we also examined whether there were non-linear associations between predicted FM and LBM and the diabetes risk in both sexes based on the fully adjusted model (model 4) using a 4-knot restricted cubic spline function. If a non-linear correlation was found, we will further search for the best inflection point on the spline regression curve using the two-piecewise linear regression model and when the two-piecewise linear regression model had the largest likelihood estimate value, the corresponding inflection point was the best; and then the difference between the linear regression models on both sides of the inflection point was compared by likelihood ratio analysis.

Sensitivity analysis: (1) Excluded participants with less than two years of follow-up, with the aim of avoiding a potential reverse causality from affecting the results. (2) We explored the possibility of unmeasured confounding between predicted LBM and FM and diabetes risk by calculating E-values (31). The E-value quantifies the required magnitude of an unmeasured confounder that could negate the observed association between predicted LBM and FM and diabetes risk.

We also performed subgroup analyses to explore the effects of age, BMI, smoking status, exercise habits, hypertension, and fatty liver on the associations between predicted FM and LBM and diabetes risk and checked for interactions between subgroups using the log-likelihood ratio test.

## Results

### Baseline characteristics

After screening by the exclusion criteria, 15,463 eligible participants were finally included in this study; among them, 8,429 were men, with an average age of 44.09 (9.00); 7,034 were women, with an average age of 43.25 (8.76). During a mean follow-up period of 6.13 years, a total of 373 diabetes cases (3.93/1,000 person-years) occurred. Table 1 presents the baseline characteristics of diabetic versus non-diabetic participants in both sexes. Obviously, there had already been significant differences in the indicators among the future diabetic and non-diabetic patients at baseline, especially the blood glucose metabolism indicators (HbA1c, FPG) had the largest difference (standardized difference > 100, all *P* < 0.001). In addition, compared to the non-diabetic group, the diabetic group had higher predicted LBM (standardized difference: 40% in women, 30% in men; *P* < 0.001) and FM (standardized difference: 90% in women, 70% in men; *P* < 0.001), age, weight, BMI, WC, TC, DBP, SBP, TG, LDL-C, GGT, AST, ALT, and more smokers and fatty liver patients, but had lower height and HDL-C levels (all *P* < 0.05); whereas in exercise habits and drinking status differed significantly only among men (all *P* < 0.05). Additionally, by comparing the standardized difference values between groups for both sexes we found that differences in almost all baseline indicators were greater in women than in men.

**TABLE 1:** Baseli Pne characteristics of participants grouped by sex and diabetes.

Characteristic	Women		Standardized difference,% (95% CI)	*P*-value	Men		Standardized difference,% (95% CI)	*P*-value
	Non-diabetic	Diabetic			Non-diabetic	Diabetic		
No. of participants	6,947	87			8,143	286		
LBM*^a^*, kg	33.8 (3.3)	35.4 (4.5)	40 (20, 60)	<0.001	50.7 (5.4)	52.8 (6.7)	30 (20, 50)	<0.001
FM*^a^*, kg	16.9 (14.3–20.1)	21.6 (18.0–26.4)	90 (70, 110)	<0.001	14.6 (11.3–17.5)	18.1 (14.2–22.2)	70 (60, 80)	<0.001
Age, year	42.0 (37.0–49.0)	47.0 (41.0–53.5)	50 (30, 70)	<0.001	42.0 (36.0–50.0)	46.0 (40.2-52.0)	30 (20, 50)	<0.001
Weight, kg	52.6 (7.8)	59.5 (11.1)	70 (50, 90)	<0.001	67.1 (9.8)	73.0 (12.3)	50 (40, 60)	<0.001
Height, cm	158.3 (5.4)	155.9 (6.5)	40 (20, 60)	<0.001	170.8 (6.0)	170.0 (6.0)	10 (0.0, 30)	0.016
BMI, kg/m^2^	21.0 (2.9)	24.5 (4.4)	90 (70, 120)	<0.001	23.0 (2.9)	25.2 (3.6	70 (60, 80)	<0.001
WC, cm	71.6 (8.0)	80.4 (11.8)	90 (70, 110)	<0.001	80.3 (7.8)	86.5 (9.2)	70 (60, 90)	<0.001
TC, mmol/L	5.1 (0.9)	5.6 (0.9)	50 (30, 70)	<0.001	5.2(0.8)	5.4 (0.9)	30 (20, 40)	<0.001
TG, mmol/L	0.6 (0.4–0.8)	1.0 (0.7-1.3)	80 (60, 100)	<0.001	0.9(0.6–1.4)	1.4 (0.9–2.1)	60 (50, 70)	<0.001
HDL-C, mmol/L	1.6 (1.4–1.9)	1.3 (1.1–1.6)	70 (50, 90)	<0.001	1.3(1.1–1.5)	1.1 (0.9–1.3)	60 (50, 70)	<0.001
DBP, mmHg	67.6 (9.8)	72.9 (9.3)	60 (30, 80)	<0.001	74.7 (9.9)	78.5 (10.2)	40 (30, 50)	<0.001
SBP, mmHg	109.3 (14.3)	117.0 (15.0)	50 (30, 70)	<0.001	118.6 (14.1)	123.5 (15.5)	30 (20, 50)	<0.001
HbA1c,%	5.2 (0.3)	5.6 (0.4)	120 (90, 140)	<0.001	5.1 (0.3)	5.5 (0.4)	110 (100, 120)	<0.001
FPG, mmol/L	5.0 (0.4)	5.5 (0.4)	120 (100, 140)	<0.001	5.3(0.4)	5.7 (0.3)	100 (90, 120)	<0.001
GGT, U/L	12.0 (10.0–15.0)	15.0 (12.0–22.5)	60 (30, 80)	<0.001	19.0 (15.0–28.0)	26.0 (19.0–39.8)	30 (20, 50)	<0.001
AST, U/L	14.0 (11.0–17.0)	19.0 (14.0–23.0)	60 (30, 80)	<0.001	18.0 (15.0–23.0)	20.5 (17.0–26.8)	60 (50, 70)	<0.001
ALT, U/L	16.0 (13.0–19.0)	18.0 (15.0–22.0)	40 (20, 60)	<0.001	20.0 (15.0–27.0)	28.0 (20.0–42.8)	40 (30, 50)	<0.001
Exercise habits, *n* (%)			10 (0.0, 30)	0.611			10 (0.0, 30)	0.019
No	5,850 (84.2%)	75 (86.2%)			6,583 (80.8%)	247 (86.4%)		
Yes	1,097 (15.8%)	12 (13.8%)			1,560 (19.2%)	39 (13.6%)		
Fatty liver, *n* (%)			100 (80, 120)	< 0.001			80 (70, 100)	<0.001
No	6,502 (93.6%)	46 (52.9%)			6,070 (74.5%)	104 (36.4%)		
Yes	445 (6.4%)	41 (47.1%)			2,073 (25.5%)	182 (63.6%)		
Smoking status, *n* (%)			20 (0.0, 50)	0.020			30 (20, 40)	<0.001
None	6,069 (87.4%)	70 (80.5%)			2,817 (34.6%)	75 (26.2%)		
Past	436 (6.3%)	5 (5.7%)			2,438 (29.9%)	72 (25.2%)		
Current	442 (6.4%)	12 (13.8%)			2,888 (35.5%)	139 (48.6%)		
Drinking status, *n* (%)			20 (0.0, 40)	0.391			20 (10, 30)	0.018
Non/Small	6,369 (91.7%)	82 (94.3%)			5,170 (63.5%)	184 (64.3%)		
Light	387 (5.6%)	2 (2.3%)			1,331 (16.3%)	38 (13.3%)		
Moderate	191 (2.7%)	3 (3.4%)			1131 (13.9%)	34 (11.9%)		
Heavy					511 (6.3%)	30 (10.5%)		

Values were expressed as mean (standard deviation) or medians (quartile interval) or n (%). LBM, lean body mass; FM, fat mass; BMI: body mass index; WC, waist circumference; TC, total cholesterol; SBP, systolic blood pressure; DBP, diastolic blood pressure; HbA1c, glycosylated hemoglobin; FPG, fasting plasma glucose; TG, triglyceride; HDL-C, high-density lipoprotein cholesterol; LDL-C, low-density lipoprotein cholesterol; ALT, alanine aminotransferase; AST, aspartate aminotransferase; GGT, gamma-glutamyl transferase. ^a^Derived from validated anthropometric prediction equations. Diabetic group included participants with measured HbA1c ≥ 6.5% or FPG ≥ 7.0 mmol/L or self-reported diabetes diagnosis during follow-up.

### Associations of predicted FM and LBM and BMI with diabetes risk

In the collinearity screening, covariates sex, weight, WC, and DBP were excluded from subsequent model adjustment, while there was also collinearity between BMI and predicted FM and LBM, thus BMI was not mutually adjusted with predicted FM and LBM in the subsequent models (Supplementary Tables 2, 3). According to the STROBE statement, we constructed four multivariate Cox regression models (Table 2). In the stepwise adjusted multivariate Cox regression models 1–4, we found that BMI was consistently and significantly positively correlated with diabetes risk, and the HRs in the fully adjusted model 4 were 1.09 and 1.08 (all *P* < 0.05) for women and men, respectively. While the effects of the body composition indicators predicted FM and LBM on the risk of future diabetes showed an opposite trend in women. In women, each kilogram increase in predicted LBM reduced the diabetes risk by 65% (HR 0.35, 95%CI 0.17, 0.71); conversely, each kilogram increase in predicted FM increased the diabetes risk by 84% (HR 1.84, 95%CI 1.26, 2.69). However, the overall effect of the association between predicted LBM and diabetes risk was not statistically significant in men (HR 0.99, 95%CI 0.94, 1.04), and only predicted FM remained a significant positive association with diabetes risk during multivariate model adjustment (HR 1.06, 95%CI 1.01, 1.11).

**TABLE 2:** Hazard ratios for incident diabetes, by predicted LBM, FM, and BMI.

	Hazard ratios (95% confidence interval)			
	Model 1	Model 2	Model 3	Model 4
**Women**				
LBM*^a^*	0.26 (0.13, 0.50)**	0.27 (0.14, 0.51)**	0.29 (0.14, 0.58)**	0.35 (0.17, 0.71)*
FM*^a^*	2.40 (1.70, 3.38)**	2.35 (1.67, 3.31)**	2.13 (1.47, 3.07)**	1.84 (1.26, 2.69)*
BMI	1.31 (1.25, 1.38)**	1.31 (1.24, 1.37)**	1.16 (1.09, 1.24)**	1.09 (1.02, 1.17)*
**Men**				
LBM*^a^*	1.00 (0.96, 1.05)	1.01 (0.96, 1.06)	0.99 (0.94, 1.04)	0.99 (0.94, 1.04)
FM*^a^*	1.15 (1.10, 1.20)**	1.14 (1.09, 1.19)**	1.09 (1.04, 1.14)**	1.06 (1.01, 1.11)*
BMI	1.24 (1.20, 1.28)**	1.24 (1.20, 1.27)**	1.13 (1.08, 1.17)**	1.08 (1.04, 1.13)**

Model 1: Age and height. Model 2: Model 1 plus drinking status, smoking status, and exercise habits. Model 3: Model 2 plus FPG, HbA1c, TC, TG, HDL-C, SBP. Model 4: Model 3 plus ALT, AST, GGT, and fatty liver. Note: Both predicted LBM and predicted FM were mutually adjusted for each other. Abbreviations as in Table 1. ^a^Derived from validated anthropometric prediction equations. *P < 0.05; **P < 0.001.

**TABLE 3:** Thresholds for predicted LBM- and FM-related diabetes risk in men.

	Diabetes (HR, 95% CI)	
	LBM*^a^*	FM*^a^*
**Fitting model by multivariable Cox regression**		
	0.99 (0.94, 1.04)	1.06 (1.01, 1.11)
**Fitting model by two-piecewise linear regression**		
The best inflection point	45.4	13.76
<Inflection point	0.83 (0.73, 0.95)	0.92 (0.84, 1.00)
>Inflection point	1.00 (0.95, 1.05)	1.10 (1.05, 1.16)

HR, hazard ratios; CI, confidence interval; other abbreviations as in Table 1. Adjusted for age, height, drinking status, smoking status, exercise habits, FPG, HbA1c, TC, TG, HDL-C, SBP, ALT, AST, GGT and fatty liver. Both predicted LBM and predicted FM were mutually adjusted for each other. ^a^Derived from validated anthropometric prediction equations.

### Sensitivity analysis

To avoid the effect of potential reverse causality, we performed the same analysis after excluding participants with less than two years of follow-up (*n* = 2,641, 17.08%), and we found that the magnitude and direction of the associations between predicted LBM, FM, and BMI and the risk of diabetes in both sexes remained stable (Supplementary Table 4). In addition, we generated an E-value to assess sensitivity to unmeasured confounding. The results showed that predicted FM and LBM were both associated with the risk of diabetes in women, and the point estimates of the E-values were 3.08 and 5.16, respectively, indicating that it was unlikely that there was an unmeasured confounding factor that could affect the stability of the results; predicted FM was linearly correlated with the risk of diabetes in men, and the point estimate of the E value was 1.43, indicating that there may be a confounding factor affecting the results, which needs further study.

### Non-linear analysis

**FIGURE 2:**
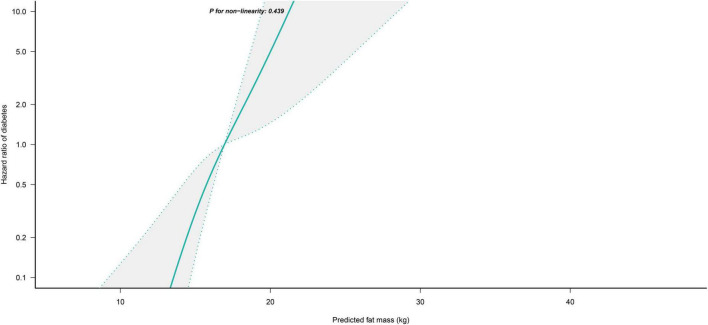
Restricted cubic spline analysis of predicted FM for the estimation of the risk of diabetes in women after adjusting for multivariate covariates. Adjusted for age, height, drinking status, smoking status, exercise habits, FPG, HbA1c, TC, TG, HDL-C, SBP, ALT, AST, GGT, LBM and fatty liver.

**FIGURE 3:**
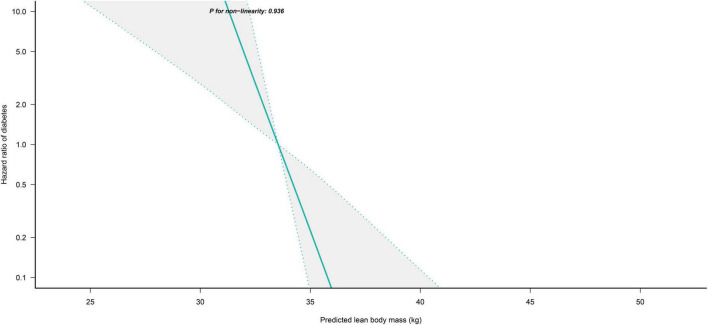
Restricted cubic spline analysis of predicted LBM for the estimation of the risk of diabetes in women after adjusting for multivariate covariates. Adjusted for age, height, drinking status, smoking status, exercise habits, FPG, HbA1c, TC, TG, HDL-C, SBP, ALT, AST, GGT, FM and fatty liver.

**FIGURE 4:**
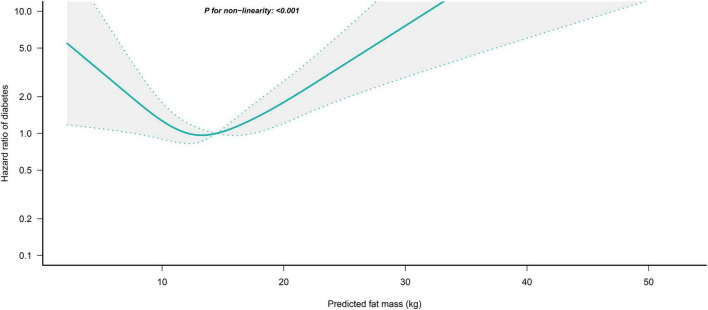
Restricted cubic spline analysis of predicted FM for the estimation of the risk of diabetes in men after adjusting for multivariate covariates. Adjusted for age, height, drinking status, smoking status, exercise habits, FPG, HbA1c, TC, TG, HDL-C, SBP, ALT, AST, GGT, LBM and fatty liver.

**FIGURE 5:**
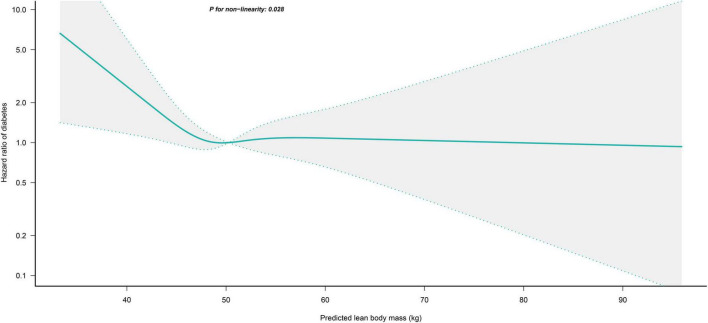
Restricted cubic spline analysis of predicted LBM for the estimation of the risk of diabetes in men after adjusting for multivariate covariates. Adjusted for age, height, drinking status, smoking status, exercise habits, FPG, HbA1c, TC, TG, HDL-C, SBP, ALT, AST, GGT, FM and fatty liver.

We further flexibly modeled the associations of predicted FM and LBM with diabetes risk in both sexes using a 4-knot restricted cubic spline function. In women, predicted FM and LBM were linearly associated with diabetes risk (all *P* for non-linear > 0.05) (Figures 2, 3), but not in men, where predicted FM was U-shaped associated with diabetes risk (*P* for non-linear < 0.001) (Figure 4), with an HR of 0.92 (0.84, 1.00) for each kilogram increase in predicted FM associated with diabetes risk when predicted FM was less than 13.76 kg, while when predicted FM was greater than 13.76 kg, the HR for that was 1.10 (1.05, 1.16) (Table 3). In addition, we also found that there was an L-shape association between predicted LBM and diabetes risk in men (*P* for non-linear = 0.028) (Figure 5), with an HR of 0.83 (0.73, 0.95) for each kilogram increase in predicted LBM associated with diabetes risk when predicted LBM was less than 45.4 kg, but no significant correlation was found when predicted LBM was greater than 45.4 kg (Table 3).

### Subgroup analysis

Table 4 shows the results of the exploratory subgroup analysis, and we found no significant effects of different age subgroups, BMI subgroups, smoking status, exercise habits, hypertension, and fatty liver on the predicted LBM- and FM-related risk of diabetes in women (all *P* for interaction > 0.05). Moreover, there were no significant interactions in men for all subgroups except for the BMI subgroups and fatty liver (Table 5). Predicted LBM was resistant to diabetes risk in men without fatty liver, despite being borderline positive; whereas predicted FM was associated with a higher risk of diabetes in men with low body weight and those with fatty liver.

**TABLE 4:** Stratified association between predicted LBM and predicted FM and diabetes by age, BMI, smoking status, and fatty liver in women.

	LBM*^a^*			FM*^a^*		
Subgroup	HR (95%CI)	*P*-value	*P* for interaction	HR (95%CI)	*P*-value	*P* for interaction
Age (years)			0.9526			0.8052
<40	0.59 (0.35, 1.01)	0.0539		1.43 (1.07, 1.91)	0.0146	
40–60	0.59 (0.35, 0.99)	0.0440		1.40 (1.05, 1.87)	0.0209	
≥60	0.57 (0.30, 1.07)	0.0800		1.38 (1.00, 1.89)	0.0473	
BMI(kg/m^2^)			0.9703			0.7473
<18.5	0.33 (0.13, 0.86)	0.0233		2.32 (0.78, 6.94)	0.1322	
18.5–25	0.36 (0.17, 0.72)	0.0045		1.78 (1.21, 2.61)	0.0033	
≥25	0.36 (0.18, 0.73)	0.0045		1.84 (1.26, 2.69)	0.0016	
Smoking status			0.5683			0.8798
None	0.34 (0.17, 0.69)	0.0030		1.84 (1.26, 2.69)	0.0015	
Past	0.39 (0.19, 0.81)	0.0117		1.94 (1.27, 2.97)	0.0024	
Current	0.35 (0.17, 0.72)	0.0042		1.86 (1.26, 2.75)	0.0020	
Fatty liver			0.2562			0.8573
No	0.33 (0.16, 0.68)	0.0025		1.83 (1.25, 2.69)	0.0019	
Yes	0.35 (0.17, 0.72)	0.0040		1.85 (1.27, 2.70)	0.0014	
Exercise habits			0.2548			0.0900
No	0.35 (0.17, 0.71)	0.0038		1.81 (1.24, 2.65)	0.0021	
Yes	0.38 (0.19, 0.79)	0.0099		1.99 (1.35, 2.93)	0.0005	
Hypertension			0.2237			0.1397
No	0.38 (0.19, 0.76)	0.0064		1.79 (1.23, 2.60)	0.0022	
Yes	0.34 (0.16, 0.69)	0.0029		1.63 (1.10, 2.42)	0.0158	

HR, hazard ratios; CI, confidence interval; other abbreviations as in Table 1. Models adjusted for the same covariates as in model 4 (Table 2), except for the stratification variable. ^a^Derived from validated anthropometric prediction equations.

**TABLE 5:** Stratified association between predicted LBM and predicted FM and diabetes by age, BMI, smoking status, and fatty liver in men.

	LBM*^a^*			FM*^a^*		
Subgroup	HR (95%CI)	*P*-value	*P* for interaction	HR (95%CI)	*P*-value	*P* for interaction
Age (years)			0.2299			0.0851
<40	0.99 (0.93, 1.05)	0.8185		1.10 (1.04, 1.16)	0.0013	
40–60	0.98 (0.93, 1.03)	0.3831		1.08 (1.02, 1.13)	0.0037	
≥60	0.91 (0.81, 1.01)	0.0796		0.97 (0.87, 1.08)	0.5570	
BMI(kg/m^2^)			0.0805			0.0260
<18.5	1.01 (0.78, 1.32)	0.9147		1.35 (0.87, 2.10)	0.1757	
18.5–25	0.94 (0.88, 1.01)	0.0723		1.00 (0.94, 1.07)	0.9407	
≥25	1.00 (0.95, 1.05)	0.9693		1.10 (1.04, 1.16)	0.0010	
Smoking status			0.2929			0.3623
None	0.98 (0.93, 1.04)	0.5597		1.06 (1.00, 1.12)	0.0625	
Past	0.97 (0.91, 1.03)	0.3567		1.03 (0.96, 1.10)	0.3773	
Current	1.01 (0.95, 1.06)	0.8050		1.08 (1.02, 1.13)	0.0041	
Fatty liver			0.0187			0.0006
No	0.95 (0.89, 1.01)	0.0935		1.00 (0.94, 1.06)	0.9561	
Yes	1.00 (0.95, 1.05)	0.9305		1.10 (1.05, 1.16)	0.0002	
Exercise habits			0.3694			0.2136
No	1.00 (0.95, 1.05)	0.8620		1.07 (1.02, 1.12)	0.0059	
Yes	0.97 (0.91, 1.04)	0.4085		1.03 (0.95, 1.11)	0.4862	
Hypertension			0.2373			0.1756
No	1.00 (0.95, 1.05)	0.9486		1.07 (1.02, 1.12)	0.0044	
Yes	0.98 (0.92, 1.03)	0.4066		1.03 (0.97, 1.10)	0.2949	

HR, hazard ratios; CI, confidence interval; other abbreviations as in Table 1. Models adjusted for the same covariates as in model 4 (Table 2), except for the stratification variable. ^a^Derived from validated anthropometric prediction equations.

## Discussion

In this large longitudinal cohort study of the Japanese general population in Asia, we investigated the relationship between BMI and predicted FM and LBM and diabetes. Overall, increasing predicted LBM significantly reduced the risk of diabetes in women, but there was a saturation effect of predicted LBM in reducing the risk of diabetes in men, with a saturation point of 45.4 kg. Furthermore, predicted FM was stronger associated with diabetes risk in women compared with BMI, while in men, predicted FM was associated with diabetes risk in a U-shaped pattern, with the lowest diabetes risk when predicted FM was 13.76 kg. The results of sensitivity analysis and subgroup analysis further confirmed the reliability of these findings. Additionally, fatty liver status and BMI significantly affected the associations between body composition and the risk of diabetes in men, with a significantly stronger association between predicted FM and diabetes in those with low body weight and fatty liver.

It is well known that obesity, one of the most important risk factors for diabetes, is closely associated with IR, abnormal glucose tolerance, and hyperinsulinemia (13, 32). BMI is currently the most commonly used simple anthropometric obesity parameter and is widely used in epidemiological studies and clinical practice for risk estimation and risk stratification of obesity-related diseases. However, the main limitation of BMI is its inability to differentiate between fat and lean muscle mass (8), and using BMI only as an obesity indicator to assess diabetes risk in the general population may result in incorrect risk estimates and inefficient risk stratification, as the athletes and the general population, as well as men and women, clearly have different diabetes risks with the same BMI. Therefore, we speculated that further exploring the association between body composition, FM and LBM, and diabetes risk on the basis of BMI might compensate for the limitation. However, DXA, the gold standard measurement method for participants’ body composition FM and LBM, has high technical requirements and economic cost and is not suitable for large-scale epidemiological studies and clinical screening of related diseases. Therefore, we calculated alternative indicators of body composition namely predicted FM and LBM using the anthropometric prediction equations with very high predictive performance (*R*^2^ > 0.9) developed and validated by Lee et al. (22).

Previous studies have shown that predicted FM and LBM were cost-effective alternative indicators of body composition which are in high agreement with the actual FM and LBM measured by DXA and can be used to analyze the impact of body composition on the risk of diseases such as all-cause and cause-specific mortality, cardiovascular disease, and lung cancer, as well as for explaining the obesity paradox (9, 33–35). However, studies investigating the relationship between body composition and diabetes risk are limited, and the results remain controversial. Evidence from experimental studies has shown that the effects of FM and LBM on the body’s glucose metabolism are significantly different. Excess body fat releases excessive amounts of pro-inflammatory cytokines, glycerol, fatty acids, and other substances that promote IR thereby increasing the risk of diabetes (13, 36). By contrast, LBM consists mainly of skeletal muscle, which is the most important organ for the uptake of glucose from human blood, accounting for approximately 85% of all insulin-mediated glucose utilization; myofibers of skeletal muscle also express and release a number of cytokines or peptides, including irisin and interleukins, which are important for maintaining insulin sensitivity of skeletal muscle cells (12, 37). Evidence from observational studies generally supports predicted FM as an independent risk factor for diabetes (10, 14–16), yet the association between predicted LBM and diabetes risk is much debated (14, 16–21, 38, 39). In the Helsinki Birth Cohort study by Rehunen SKJ et al. it was found that LBM had no significant effect on glucose metabolism in people without excess body fat mass, whereas more LBM in people who were overweight or obese posed a higher risk of type 2 diabetes (17); similarly, in a study by Liu et al., they also found predicted LBM was a risk factor for type 2 diabetes among men (16). But considering the small sample sizes of 704 and 687 participants in the studies by Rehunen et al. and Liu et al., respectively, and the fact that neither of them adjusted for FM when studying the association between LBM and diabetes risk, these may cause biased risk estimates. Overweight or obese individuals naturally require more LBM to carry the weight load, and the increased risk of diabetes in the state of high FM and high LBM may be due to the fact that the harms of high FM outweigh the benefits of high LBM (17). In a retrospective cohort study by Baker et al. which included 54,295 Danish participants, LBM was found to significantly increase the risk of type 2 diabetes before adjustment for FM, but when they further adjusted for FM they found that LBM was negatively associated with type 2 diabetes in men (14). Furthermore, in studies by Hong et al. and Srikanthan et al. relative muscle mass was found to be negatively associated with impaired fasting glucose and the risk of type 2 diabetes, and maintaining and boosting muscle mass may be important to prevent type 2 diabetes (38, 39).

In the current study, for investigating the effects of separate predicted FM and LBM on the onset of diabetes, we adjusted predicted FM and LBM for each other in all models. Interestingly, our findings suggested that the independent effect of body composition on diabetes risk in women was stable, while the effect of that in men was variable. In women, each kilogram increase in predicted LBM reduced the diabetes risk by 65%, and the association of predicted FM with diabetes risk was also significantly stronger than that of BMI (HR: 1.84 vs. 1.09). Additionally, although BMI was more strongly associated with diabetes risk than predicted FM and LBM in men, we found non-linear associations between predicted FM and LBM and diabetes risk. There was a U-shaped association between predicted FM and diabetes risk in men, with the lowest risk of developing diabetes when the predicted FM was equal to 13.76 kg, and an L-shaped association between predicted LBM and diabetes risk in men, with an increase in predicted LBM helping to reduce diabetes risk when the predicted LBM in men was less than 45.4 kg, but no significant effect on diabetes risk when it was higher than 45.4 kg. These results may be caused by differences in body fat distribution between men and women and the strong correlation between BMI and WC (40, 41). Men and women have different fat deposition patterns due to differences in sex hormone levels; women tend to store fat in the hips and thighs, while men tend to store fat in the abdominal subcutaneous and visceral organs (28, 40, 42). Abdominal obesity is known to be an important risk factor and a typical sign of IR and diabetes, and thus, fat distribution patterns in men other than FM mediate a significant portion of obesity-related diabetes risk, whereas relatively healthy fat distribution in women does not produce additional diabetes risk. Moreover, a strong correlation between BMI and the abdominal obesity indicator WC was demonstrated in the study of Christakoudi S et al. (*r* = 0.8–0.9) (41), implying that BMI can also partially explain changes in abdominal fat distribution. Therefore, women needed to focus more on the impact of body composition indicators on diabetes risk; but in men, the good correlation between BMI and central obesity makes its association with diabetes risk stronger than predicted FM. In addition, in the restricted cubic spline analysis, we found a U-shaped association between the predicted FM and the diabetes risk in men, which may also explain the weak overall effect size of the association between predicted FM and diabetes risk in men. When the predicted FM was less than 13.76 kg it was negatively correlated with the diabetes risk in men, which may be related to the uptake and storage of harmful free fatty acids in the circulation by adipose tissue (43). Previous studies have shown that free fatty acids in peripheral insulin-sensitive tissues can induce IR through multiple mechanisms (44, 45) and that peripheral upper body subcutaneous fat, but not abdominal visceral fat, is the primary tissue for uptake and storage of harmful free fatty acids throughout the body (46, 47). Therefore, men need to maintain a moderate amount of fat while controlling their diet and losing weight to reduce the risk of developing diabetes.

As in previous studies, our results also revealed that predicted FM in women was a more important risk factor for diabetes than BMI; however, the difference is that we found for the first time that predicted LBM was a strong protective factor for diabetes in women, and our results remained stable after validation by sensitivity analysis and multiple subgroup analysis. This may be related to the mutual adjustment of the predicted FM and LBM in the current study and the fact that the women in this study were mainly middle-aged. Previous studies have shown that overall body weight in both sexes may keep the same or increase only slightly before reaching old age, but the proportions of FM and LBM will change markedly from midlife, especially in women (48). In terms of LBM, the natural decline in skeletal muscle mass with aging follows different trajectories in men and women after middle age, with men tending to have a gradual decline in skeletal muscle; whereas women have a rapid decline in both skeletal muscle mass and function after menopause, which could lead to a dramatic reduction in the ability of the skeletal muscle to take up blood glucose and a decrease in insulin sensitivity (49). Therefore, postmenopausal women are more prone to uncompensated increases in blood glucose and even diabetes (12, 37). In terms of FM, the total FM of men may increase slightly but the deposition pattern of fat does not change, while the rapid decline of estrogen levels in women after menopause causes more deposition of newly generated adipose tissue in the abdominal subcutaneous or visceral organs, resulting in abdominal obesity and IR (50). Thus, the combined effects of aging and menopause cause the rapid decline of LBM in women and the ectopic deposition of adipose tissue, which will lead to the dysfunction of blood glucose regulation and the occurrence of IR. In addition, although the Cox regression analysis did not find a protective effect of predicted LBM on the diabetes risk in men, in further spline regression analysis we found that the predicted LBM was L-shape associated with the diabetes risk (Figure 3), and when the predicted LBM was less than 45.4 kg it was significantly negatively correlated with diabetes risk in men; thus, the progressive decline in skeletal muscle mass in men after middle age also contributes to the increased risk of diabetes in middle-aged and older men, which is consistent with the findings of Kalyani et al. who found that relatively low LBM was associated with an increased risk of diabetes in men with increasing age (21). In sum, our study showed that predicted FM and LBM had significant independent effects on diabetes risk in both sexes, especially in women; consequently, to prevent diabetes we recommend that women should increase physical activity and appropriate muscle-strengthening training in addition to reducing fat intake through diet control, whereas men should control their diet and lose weight while keeping their FM around to 13.76 kg and their LBM above 45.4 kg. However, it is worth noting that the 25th percentile, median, and 75th percentile values of predicted LBM for men in the current study were 47.0, 50.3, and 53.9 kg, respectively, so >75% of men participants in the current cohort had a predicted LBM higher than 45.4 kg, which may imply that most Asian men have less benefit in reducing their risk of diabetes by increasing their LBM. In addition, the 25th percentile, median, and 75th percentile values of predicted FM for men in the current cohort were 11.3, 14.4, and 17.7 kg, respectively; thus, >50% of men participants had a predicted FM higher than 13.76 kg, implying that lowering FM levels may be effective in reducing the risk of diabetes in most Asian men. In summary, we believed that Asian men should actively reduce their fat intake to minimize the risk of diabetes, based on the prevention of aging-related LBM loss.

### Advantages and limitations

The current study had several advantages: (1) Our long follow-up period and large sample size compared to similar studies gave us more adequate statistical efficacy. (2) After a rigorous study design and repeated statistical demonstrations, we found for the first time that predicted LBM was a protective factor for diabetes in women and in men with predicted LBM of less than 45.4 kg, which provided important reference material for diabetes prevention and interventions.

Of course, this study also had some limitations: (1) The predicted FM and LBM calculated by the anthropometric prediction equations in this study as alternative indicators of body composition cannot fully and accurately represent the actual FM and LBM. In addition, these predictive equations were developed and validated by Lee et al. in two cohorts of the NHANES database with predominantly US populations, rather than specifically for Asian populations. Although race was considered in these equations, i.e., the differential effects of Mexican American, Hispanic, Black, and Other races, and the equations have been used in previous studies to calculate body compositions in Asian populations (16, 51), it is important to note that Other races were represented in only about 4% of the development and validation cohort by Lee et al. and that Other races were not clearly defined; the inclusion of a larger range of other races, of which the Asian population may be only a fraction, may prevent their equations from estimating the body composition of participants in the current study cohort as accurately as they did for the US population (*R*^2^ > 0.9). Overall, although the anthropometric prediction equations in the current study are cost-effective tools for assessing body compositions, they were not developed in Asian populations, and there may be significant differences in body compositions across ethnic groups, so the applicability of these anthropometric prediction equations to Asian populations requires additional studies for validation, and the results of the current study should be used with caution. (2) The main outcome in the current study was the onset of diabetes, but the diagnostic criteria did not include two-hour postprandial glucose values, which may underestimate the prevalence of diabetes in the current study cohort and thus the extent to which body composition indicators were associated with diabetes risk; in addition, deaths during follow-up were not recorded, which may pose a competing risk to the current findings. (3) Since the current study is a retrospective cohort study, there may be some data on diabetes risk factors that cannot be obtained, resulting in residual confounding; However, in the current study, we further calculated the E-value to quantify the potential impact of unmeasured confounding factors. The results showed that the direct correlation between predicted FM and LBM and diabetes risk in women was stable, while the direct correlation between predicted FM and LBM and diabetes in men may be affected by an unmeasured confounding factor, which needs further study. (4) Since the current study did not differentiate the types of diabetes (type 1 diabetes, type 2 diabetes, gestational diabetes, and other specific types of diabetes) that occurred during the follow-up period, it cannot be clearly stated that the analysis of the current study was for type 2 diabetes, but considering that IR caused by obesity is mainly the pathological basis of type 2 diabetes and more than 90% of diabetes is type 2 diabetes (2, 13), our results may be more applicable to type 2 diabetes.

## Conclusion

Predicted LBM was a protective factor for diabetes and predicted FM was a more important risk factor for diabetes than BMI in Asian women. But, in men, the protective effect of predicted LBM on the risk of diabetes had a saturating effect, and continued increases in predicted LBM above 45.4 kg may not further reduce the risk of diabetes; in addition, we found a U-shaped association between predicted FM and diabetes risk, with the lowest risk of diabetes when the predicted FM reached 13.76 kg. Therefore, taking into account the distribution of LBM and FM levels in both sexes in the current study population, we suggested that Asian adult women should add appropriate muscle-strengthening exercises to traditional lipid-lowering interventions to further reduce the risk of future diabetes, while Asian adult men should actively reduce their fat intake to prevent diabetes based on the prevention of aging-induced LBM loss.

## Data availability statement

The original contributions presented in the study are included in the article/Supplementary material, further inquiries can be directed to the corresponding author.

## Ethics statement

The studies involving human participants were reviewed and approved by the Ethics Committee of Jiangxi Provincial People’s Hospital. Written informed consent for participation was not required for this study in accordance with the national legislation and the institutional requirements.

## Author contributions

YZ revised the manuscript and designed the study. All authors conceived the research, drafted the manuscript, did the statistical analysis, and read and approved the final manuscript.
