# Supplementary material for: Association of predicted fat mass and lean body mass with diabetes: a longitudinal cohort study in an Asian population
Source: Front Nutr. 2023 May 9;10:1093438. doi: 10.3389/fnut.2023.1093438 (PMC10203423; doi:10.3389/fnut.2023.1093438)
Supplement: Supplementary file 1 [file Data_Sheet_1.docx]

Supplementary Table 1. Anthropometric prediction equations for predicted LBM and FM developed from the National Health and Nutrition Examination Survey.

| **LBM** |
| --- |
| **Men**  19.363 + 0.001 * age (year) + 0.064 * height (cm) + 0.756 * weight (kg) − 0.366 * waist circumference (cm) − 1.007 |
| **Women**  −10.683 − 0.039 * age (year) + 0.186 * height (cm) + 0.383 * weight (kg) − 0.043 * waist circumference (cm) − 0.340 |
| **FM** |
| **Men**  −18.592−0.009*age (year)−0.080*height (cm) + 0.226 * weight (kg) + 0.387 * waist circumference (cm) + 1.050 |
| **Women**  11.817+0.041 * age (year) − 0.199 * height (cm) + 0.610 * weight (kg) + 0.044 * waist circumference (cm) + 0.325 |

Abbreviations as in Table 1.

Supplementary Table 2: Collinearity diagnostics steps of predicted LBM and FM with other covariates.

|  | Variance inflation factor | | | | | |
| --- | --- | --- | --- | --- | --- | --- |
|  | Step 1 | Step 2 | Step 3 | Step 4 | Step 5 | Step 6 |
| LBM | 55902.4 | 117.8 | 35.2 | 8.9 | 6.6 | 6.6 |
| FM | 14674.8 | 80.4 | 18 | 3.1 | 1.3 | 1.3 |
| Sex | 46.7 | 46.5 | 37.6 | NA | NA | **NA** |
| Age | 2.4 | 1.5 | 1.5 | 1.4 | 1.3 | 1.3 |
| Height | 89.8 | 50.9 | 6.1 | 4.6 | 4.5 | 4.5 |
| Weight | 81301 | NA | NA | NA | NA | **NA** |
| BMI | 149.4 | 112.4 | NA | NA | NA | **NA** |
| WC | 11.8 | 11.8 | 11.3 | 6.4 | NA | **NA** |
| Exercise | 1 | 1 | 1 | 1 | 1 | 1 |
| ALT | 4.2 | 4.2 | 4.2 | 4.1 | 4.1 | 4.1 |
| AST | 3.3 | 3.3 | 3.3 | 3.3 | 3.3 | 3.3 |
| GGT | 1.5 | 1.5 | 1.5 | 1.5 | 1.5 | 1.5 |
| HDL-C | 1.8 | 1.8 | 1.8 | 1.8 | 1.8 | 1.8 |
| TC | 1.5 | 1.4 | 1.4 | 1.4 | 1.4 | 1.4 |
| TG | 1.8 | 1.8 | 1.8 | 1.8 | 1.8 | 1.8 |
| FPG | 1.5 | 1.5 | 1.5 | 1.5 | 1.5 | 1.5 |
| HbA1c | 1.3 | 1.3 | 1.3 | 1.3 | 1.3 | 1.2 |
| Drinking status | 1.3 | 1.3 | 1.3 | 1.3 | 1.3 | 1.3 |
| Smoking status | 1.4 | 1.4 | 1.4 | 1.4 | 1.4 | 1.4 |
| SBP | 5.6 | 5.6 | 5.6 | 5.6 | 5.6 | 1.4 |
| DBP | 5.7 | 5.7 | 5.7 | 5.7 | 5.7 | **NA** |
| Fatty liver | 1.6 | 1.6 | 1.6 | 1.6 | 1.6 | 1.6 |

Note-1: Variance inflation factor = 1/(1-R^2^). Abbreviations as in Table 1.

Note-2: The variables with Variance inflation factor >5 will be regarded as collinear variables and cannot be included in the multiple regression model.

Supplementary Table 3: Collinearity diagnostics steps of BMI with other covariates.

|  | Variance inflation factor | | | | | |
| --- | --- | --- | --- | --- | --- | --- |
|  | Step 1 | Step 2 | Step 3 | Step 4 | Step 5 | Step 6 |
| LBM | 55902.4 | 117.8 | NA | NA | NA | **NA** |
| FM | 14674.8 | 80.4 | 56.1 | NA | NA | **NA** |
| Sex | 46.7 | 46.5 | 46.3 | 3.2 | 3.2 | 3.2 |
| Age | 2.4 | 1.5 | 1.5 | 1.4 | 1.3 | 1.3 |
| Height | 89.8 | 50.9 | 6.9 | 2.8 | 2.4 | 2.4 |
| Weight | 81301 | NA | NA | NA | NA | **NA** |
| BMI | 149.4 | 112.4 | 33.6 | 5.1 | 1.8 | 1.8 |
| WC | 11.8 | 11.8 | 11.7 | 6 | NA | **NA** |
| Exercise | 1 | 1 | 1 | 1 | 1 | 1 |
| ALT | 4.2 | 4.2 | 4.1 | 4.1 | 4.1 | 4.1 |
| AST | 3.3 | 3.3 | 3.3 | 3.3 | 3.3 | 3.3 |
| GGT | 1.5 | 1.5 | 1.5 | 1.5 | 1.5 | 1.5 |
| HDL-C | 1.8 | 1.8 | 1.8 | 1.8 | 1.8 | 1.8 |
| TC | 1.5 | 1.4 | 1.4 | 1.4 | 1.4 | 1.4 |
| TG | 1.8 | 1.8 | 1.8 | 1.8 | 1.8 | 1.8 |
| FPG | 1.5 | 1.5 | 1.5 | 1.5 | 1.5 | 1.5 |
| HbA1c | 1.3 | 1.3 | 1.3 | 1.3 | 1.3 | 1.2 |
| Drinking status | 1.3 | 1.3 | 1.3 | 1.3 | 1.3 | 1.3 |
| Smoking status | 1.4 | 1.4 | 1.4 | 1.4 | 1.4 | 1.4 |
| SBP | 5.6 | 5.6 | 5.6 | 5.6 | 5.6 | 1.4 |
| DBP | 5.7 | 5.7 | 5.7 | 5.7 | 5.7 | **NA** |
| Fatty liver | 1.6 | 1.6 | 1.6 | 1.6 | 1.6 | 1.6 |

Note-1: Variance inflation factor = 1/(1-R^2^). Abbreviations as in Table 1.

Note-2: The variables with Variance inflation factor >5 will be regarded as collinear variables and cannot be included in the multiple regression model.

Supplemental Table 4: Hazard ratios for incident diabetes, by predicted LBM, FM, and BMI in population excluding participants with less than 2 years of follow-up.

|  | Hazard ratios (95% confidence interval) | | | |
| --- | --- | --- | --- | --- |
|  | Model 1 | Model 2 | Model 3 | Model 4 |
| Women |  |  |  |  |
| LBM^&^ | 0.22 (0.11, 0.45)^**^ | 0.23 (0.12, 0.48)^**^ | 0.26 (0.12, 0.55)^**^ | 0.30 (0.14, 0.65)^*^ |
| FM^&^ | 2.62 (1.81, 3.81)^**^ | 2.55 (1.76, 3.69)^**^ | 2.29 (1.54, 3.40)^**^ | 2.03 (1.35, 3.07)^**^ |
| BMI | 1.34 (1.27, 1.41)^**^ | 1.34 (1.27, 1.41)^**^ | 1.20 (1.12, 1.29)^**^ | 1.12 (1.04, 1.22)^**^ |
| Men |  |  |  |  |
| LBM^&^ | 1.01 (0.96, 1.06) | 1.01 (0.96, 1.07) | 1.00 (0.95, 1.05) | 1.00 (0.95, 1.06) |
| FM^&^ | 1.15 (1.10, 1.20)^**^ | 1.14 (1.09, 1.20)^**^ | 1.09 (1.04, 1.15)^**^ | 1.06 (1.01, 1.12)^*^ |
| BMI | 1.26 (1.22, 1.30)^**^ | 1.25 (1.21, 1.29)^**^ | 1.14 (1.09, 1.19)^**^ | 1.10 (1.05, 1.15)^**^ |

Model 1: Age and height.

Model 2: Model 1 plus drinking status, smoking status, and exercise habits.

Model 3: Model 2 plus FPG, HbA1c, TC, TG, HDL-C, SBP.

Model 4: Model 3 plus ALT, AST, GGT, fatty liver.

#Both predicted LBMI and predicted FMI were mutually adjusted for each other. Abbreviations as in Table 1

^*^*P*<0.05; ^**^*P*<0.001; & Derived from validated anthropometric prediction equations.
